# ﻿Taxonomic clarification and lectotype designation for *Cryphalusstriatulus* Mannerheim, 1853 (non Browne, 1978, nec Browne, 1981) (Coleoptera, Curculionidae, Scolytinae), and notes on pervasive homonymy

**DOI:** 10.3897/zookeys.1183.107660

**Published:** 2023-11-02

**Authors:** Matteo Marchioro, Andrew J. Johnson, Laura Besana, Michail Yu. Mandelshtam, Massimo Faccoli, Enrico Ruzzier

**Affiliations:** 1 Department of Agronomy, Food, Natural Resources, Animals and Environment (DAFNAE), Università degli Studi di Padova, Legnaro (PD), 35020, Italy Università degli Studi di Padova Legnaro Italy; 2 School of Forest, Fisheries, and Geomatics Sciences, University of Florida, Gainesville, Florida 32611 and Florida State Collection of Arthropods, FDACS-DPI, Gainesville, 32611, Florida, USA University of Florida Gainesville United States of America; 3 Department of Forest Protection, Wood Science and Game Management, Saint-Petersburg State Forest Technical University named after S.M. Kirov, Institutskii per., 5, 194021 Saint-Petersburg, Russia Saint-Petersburg State Forest Technical University named after S.M. Kirov Saint-Petersburg Russia; 4 Department of Science, Università Roma Tre, viale G. Marconi 446, 00146 Rome, Italy Università Roma Tre Rome Italy

The bark beetle *Cryphalusstriatulus* Mannerheim, 1853 was described on the basis of two specimens from Alaska, one under the bark of unknown tree and one caught in flight (Mannerheim 1853). Schwarz (1895) listed *C.striatulus* as feeding on the spruce tree *Piceaenglemanni* Engelm. [sic!] (Pinaceae) based on specimens collected while on vacation near Alta, Cottonwood Canyon, Utah, in June 1891. These specimens share the approximate collection data (with year and host omitted) as the specimen which became the type of *Cryphalusruficollis* Hopkins, 1915. It is unclear why Hopkins treated them as such, but all subsequent publications before 2021 refer to Mannerheim’s species as *Procryphalus* Hopkins,1915 then as *Trypophloeus* Fairmaire, 1864. Schwarz also collected specimens of *Trypophloeus* from the same locality, eventually becoming the holotype of *Trypophloeuspunctipennis* Hopkins, 1915, now a synonym of *Trypophloeusnitidus* Swaine, 1912.

Later reviews of North American Cryphalini (sensu Wood, 1954) found that there was a proliferation of described names, many of which represent synonymous taxa (Wood 1954). For *Cryphalusstriatulus* Mannerheim, 1853, the identity was uncertain because the types could not be found in Mannerheim’s collection. Despite this, Wood proposed that *Trypophloeusnitidus* Swaine, 1912 was junior synonym of *Cryphalusstriatulus*, thus providing the *Trypopholeusstriatulus* [sic!] combination (Wood 1969, 1973). This change was probably based on two major features: (1) Mannerheim’s statement that the specimen was similar to *Trypophloeusgranulatus* (Ratzeburg, 1837) (Mannerheim 1853), and (2) *Trypophloeusnitidus* was the only North American Cryphalini described as being north of British Columbia in literature at that time (Wood 1969, 1973). This taxonomic combination remained unchanged until Kvamme et al. (2021) in which the authors, while looking for *Trypophloeus* types, discovered a single specimen of *Cryphalusstriatulus* Mannerheim, 1853 in the type material present in the Zoological Institute (St. Petersburg, Russia) (Fig. 1A). Kvamme et al. found that the species described by Mannerheim was a species of *Cryphalus* Erichson, 1836 and re-established the original combination (Kvamme et al. 2021), putting *Cryphalusruficollis* Hopkins, 1915 as a junior synonym.

**Figure 1.**
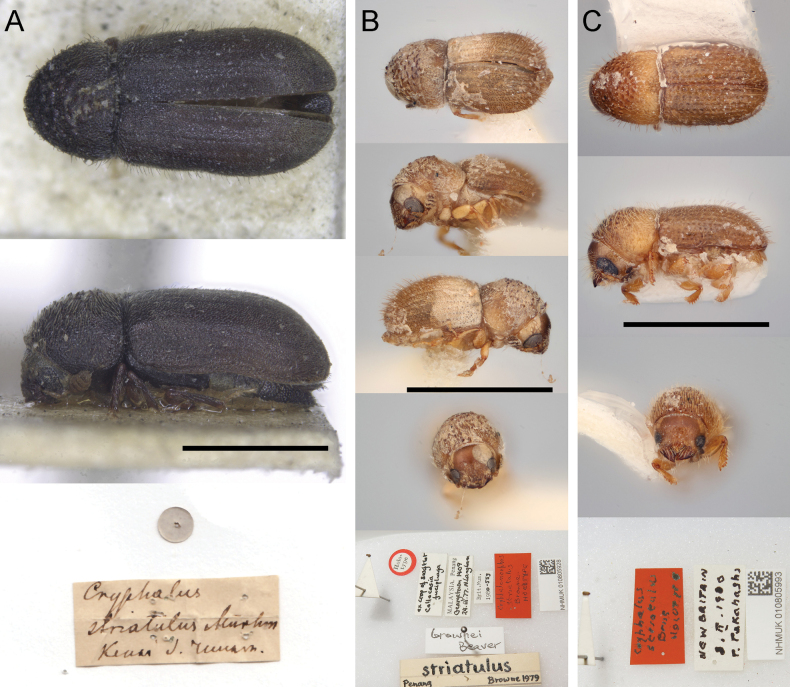
Habitus and original labels of primary types of species sharing the combination “*Cryphalusstriatulus*”. Specimen photographs are resized to a common scale, black bars represent 1.0 mm **A***Cryphalusstriatulus* Mannerheim, 1853; designated lectotype, Zoological Institute (St. Petersburg, Russia) **B***Cryphalusbrownei* (Beaver, 1991) (= *Cryphalomorphusstriatulus* Browne, 1978) holotype, NHMUK 010805928 **C***Cryphaluspunctistriatulus* Johnson, 2020 (= *Cryphalusstriatulus* Browne, 1981) holotype, NHMUK 010805993.

Mannerheim (1853) explicitly described two ‘forms’ in the description, with differences that are neither diagnostic of *Cryphalus* nor *Trypophloeus*. However, given the multiple specimens with different morphologies in the original description, there remains an unlikely but possible chance that the source of historical confusion was that two species were present in the type series. To maintain taxonomic stability, we designate the only known co-type as the lectotype of *Cryphalusstriatulus*. This specimen bears the labels: (1) golden circle used to mark type specimens; (2) “*Cryphalusstriatulus* Mnrhm. Kenai J[unio]. [?] Russam [?Russian America]” (unclear); and (3) “Lectotype *Cryphalusstriatulus* Mnrhm. Marchioro et al. des. 2023”. This designation promotes stability in case the second specimen is found and represents a different species.

Additionally, the taxonomic change following the discovery of the type creates a new homonym. *Cryphalusstriatulus* (Browne, 1978) was originally described as *Cryphalomorphusstriatulus* Browne, 1978 on the basis of a single specimen from Penang, Malaysia (Fig. 1B), collected in the gut of a swiftlet (Beaver and Browne 1978). Subsequently, the species was moved to *Hypothenemus* Westwood, 1834 and given the replacement name *Hypothenemusbrownei* Beaver, 1991 since this taxonomic combination was already pre-occupied by *Hypothenemusstriatulus* Schedl, 1942 (Schedl 1942; Beaver 1991). In 2020, Johnson et al. removed the species from *Hypothenemus* and placed it in *Cryphalus*, rendering the replacement name no longer necessary, using *Cryphalusstriatulus* (Browne, 1978) as the valid combination (Johnson et al. 2020). Since the combination *Cryphalusstriatulus* is now occupied by *Cryphalusstriatulus* Mannerheim, 1853 (sensu Kvamme et al. 2021), we treat the name *Cryphalusstriatulus* (Browne, 1978) as a junior homonym, and the novel combination, *Cryphalusbrownei* (Beaver, 1991), as the valid name. This is an unusual nomenclatural situation, for using a replacement name as a valid name when in a different genus.

An additional potential source of confusion is *Cryphalusstriatulus* Browne, 1981 (Fig. 1C), which was already replaced with *Cryphaluspunctistriatulus* Johnson, 2020 due to homonymy with *Cryphalusstriatulus* (Browne, 1978) (Johnson et al. 2020). Furthermore, *Cryphalusbrownei* Wood, 1992, already exists as an unnecessary replacement name for *Cryphalusartocarpus* Schedl, 1958, (now a synonym of *Cryphalusartocarpus* (Schedl, 1939)) (Johnson et al. 2020). Given such a high degree of homonymy within *Cryphalus*, we suggest correctly citing the authority with the year to avoid future confusion.

## ﻿Additional information

### Author ORCIDs

Matteo Marchioro https://orcid.org/0000-0003-2301-1047

Andrew J. Johnson https://orcid.org/0000-0003-3139-2257

Laura Besana https://orcid.org/0009-0004-4670-5775

Michail Y. Mandelshtam https://orcid.org/0000-0002-7135-3239

Massimo Faccoli https://orcid.org/0000-0002-9355-0516

Enrico Ruzzier https://orcid.org/0000-0003-1020-1247

### Data availability

All of the data that support the findings of this study are available in the main text.

## References

[B1] Beaver RA (1991) Reclassification of some Oriental and Australasian Scolytidae (Coleoptera). Entomologist’s Monthly Magazine 127: 53–54.

[B2] Beaver RA Browne FG (1978) The Scolytidae and Platypodidae (Coleoptera) of Penang, Malaysia. Oriental Insects 12(4): 575–624. 10.1080/00305316.1978.10432538

[B3] Hopkins AD (1915) Classification of the Cryphalinae with descriptions of new genera and species. United States Department of Agriculture: Report 99. 10.5962/bhl.title.65905

[B4] Johnson AJ Hulcr J Knížek M Atkinson TH Mandelshtam MY Smith SM Cognato AI Park S Li Y Jordal BH (2020) Revision of the bark beetle genera within the former Cryphalini (Curculionidae: Scolytinae). Insect Systematics and Diversity 4(3): 1. 10.1093/isd/ixaa002

[B5] Kvamme T Mandelshtam M Salnitska M Ojeda DI (2021) A new cryptic *Trypophloeus* Fairmaire, 1864 species in Northern Fennoscandia (Coleoptera, Curculionidae) revealed by DNA analyses. Norwegian Journal of Entomology 68: 44–66.

[B6] Mannerheim CG (1853) Dritter Nachtrag zur Käfer-Fauna der nord-amerikanischen Länder des Russischen Reiches. Bulletin de la Société Impériale des Naturalistes de Moscou 26(3): 95–273.

[B7] Schedl KE (1942) Neue Scolytidae aus Java. 76. Beitrag zur morphologie und systematik der Scolytoidea. Tijdschrift voor Entomologie 85: 1–49.

[B8] Schwarz EA (1895) Note on *Hylesinussericeus*. Insect Life 7: 254–256.

[B9] Wood SL (1954) A revision of North American Cryphalini (Scolytidae, Coleoptera). The University of Kansas Science Bulletin 36: 959–1089.

[B10] Wood SL (1969) New synonymy and records of Platypodidae and Scolytidae (Coleoptera). The Great Basin Naturalist 29(3): 113–128. 10.5962/bhl.part.17054

[B11] Wood SL (1973) New synonymy in American bark beetles (Scolytidae: Coleoptera). Part III. The Great Basin Naturalist 33(3): 169–188. 10.5962/bhl.part.28154

